# Impact of Induced Moods, Sensation Seeking, and Emotional Contagion on Economic Decisions Under Risk

**DOI:** 10.3389/fpsyg.2021.796016

**Published:** 2022-01-05

**Authors:** Kirill Efimov, Ioannis Ntoumanis, Olga Kuskova, Dzerassa Kadieva, Ksenia Panidi, Vladimir Kosonogov, Nina Kazanina, Anna Shestakova, Vasily Klucharev, Iiro P. Jääskeläinen

**Affiliations:** ^1^International Laboratory of Social Neurobiology, Institute for Cognitive Neuroscience HSE University, Moscow, Russia; ^2^School of Psychological Science, University of Bristol, Bristol, United Kingdom; ^3^Brain and Mind Laboratory, Department of Neuroscience and Biomedical Engineering, Aalto University School of Science, Espoo, Finland

**Keywords:** mood induction, framing effect, sensation seeking, emotional contagion, financial risk taking, sadness, joy

## Abstract

In addition to probabilities of monetary gains and losses, personality traits, socio-economic factors, and specific contexts such as emotions and framing influence financial risk taking. Here, we investigated the effects of joyful, neutral, and sad mood states on participants’ risk-taking behaviour in a simple task with safe and risky options. We also analysed the effect of framing on risk taking. In different trials, a safe option was framed in terms of either financial gains or losses. Moreover, we investigated the effects of emotional contagion and sensation-seeking personality traits on risk taking in this task. We did not observe a significant effect of induced moods on risk taking. Sad mood resulted in a slight non-significant trend of risk aversion compared to a neutral mood. Our results partially replicate previous findings regarding the presence of the framing effect. As a novel finding, we observed that participants with a low emotional contagion score demonstrated increased risk aversion during a sad mood and a similar trend at the edge of significance was present in high sensation seekers. Overall, our results highlight the importance of taking into account personality traits of experimental participants in financial risk-taking studies.

## Introduction

In economic tasks, humans are not payoff maximisers who would strictly behave according to expected monetary outcomes (Kahneman and Tversky, 1979; Opaluch and Segerson, 1989; Henrich et al., 2005). Rather, financial risk taking is influenced by personality traits (Llewellyn, 2008), the individuals’ mood (Kusev et al., 2017), how the decisions are framed (Tversky and Kahneman, 1981; Steiger and Kühberger, 2018), as well as other demographic or socio-economic factors and specific contexts (Zuckerman and Kuhlman, 2000; Henrich et al., 2005; Eckel and Grossman, 2008). This makes human factors in financial risk taking an important area of investigation.

Emotional context has been shown to regulate neural circuits associated with proactive or passive behaviour, and as a result, influences risk assessment and risk-taking decisions (Engelmann and Hare, 2018). In particular, the positively valanced emotion of joy has been found to increase one’s tendencies for risky behaviour (Schulreich et al., 2014; Stanton et al., 2014), and high happiness has not been associated with avoidance of frequent losses compared to unhappiness (Yechiam et al., 2016). Sadness is traditionally considered to decrease risk taking (Yuen and Lee, 2003; Stanton et al., 2014; Hareli et al., 2021).

However, some studies reported conflicting results; for example, Raghunathan and Pham (1999) argued that, since the distinctive meaning structure underlying sadness is the loss or absence of a reward, sad individuals tend to pursue high-risk/high-reward options. In another instance, Yuen and Lee (2003) did not find significant differences in risk taking between people feeling joy and people in the neutral mood.

Cognitive biases can also influence financial risk taking (Zindel et al., 2014). One of the most persistent cognitive biases observed is the effect of framing according to which framing of options in terms of either potential gains or losses can influence one’s inclination to risk. Specifically, gain framing has been linked to risk aversion and loss framing to risk seeking (Tversky and Kahneman, 1981; Isen et al., 1988; Lee and Andrade, 2014; Stanton et al., 2014). The framing effect has been widely studied in various experimental settings and consistently observed; for example, in the case of framing by manipulation of reference points (Steiger and Kühberger, 2018). Stanton et al. (2014) reported no influence of sad or joyful context on the framing effect. However, Cassotti et al. (2012) observed elimination of the framing effect after participants’ exposure to emotionally pleasant photographs.

One reason for this heterogeneity in previously reported results may be the fact that the emotional effects of stimuli used to induce a particular mood may depend heavily on the individual degree of sensation seeking (SS) and emotional contagion.

Sensation seeking is a personality trait that conveys individual predisposition for seeking and undergoing intense sensory experiences (Zuckerman, 1979a). The positive relationship between risk perception/risk taking and SS has been widely established (Franken et al., 1992; Horvath and Zuckerman, 1993). People scoring high on the SS scale tend to take more risks across various domains, be it the financial domain (Wong and Carducci, 1991), health domain (Zuckerman, 1988; Desrichard and Denarié, 2005; MacPherson et al., 2010), social domain (Roberti, 2004; Desrichard and Denarié, 2005; Khodarahimi, 2015), recreational domain (Pizam et al., 2004), or ethical domain (Khodarahimi, 2015).

Emotional contagion is defined as “a tendency to automatically mimic and synchronise expressions, vocalisations, postures, and movements with those of another person’s and, consequently, to converge emotionally” (Hatfield et al., 1993). Although it is suggested that one’s emotional contagion is linked to the degree to which risk information affects them (Loewenstein et al., 2001), robust empirical evidence is missing for this proposal. Recently, this relationship has been investigated in the context of the COVID-19 pandemic showing an increase in the number of preventive measures (e.g., frequency of washing hands) among people with high emotional contagion (Jin et al., 2020).

In the present study, we aimed to investigate the effects of induced mood state on the overall propensity to gamble as well as on the framing effect, taking into account the individual degree of SS and emotional contagion. In particular, we employed an experimental paradigm similar to Stanton et al. (2014), but used a within-subjects instead of between-subjects design with Russian participants measuring the degree of SS and emotional contagion by means of well-known questionnaires (Zuckerman, 1979b; Doherty, 1997). We confirm Stanton et al.’s (2014) results, finding that the induced emotional state did not affect the significant framing effect, both for groups of participants with low and high levels of SS and groups with low and high emotional contagion. However, the observed effects of emotions on risk-taking propensity were different. Contrary to our hypothesis, joyful mood did not cause changes in risk taking, while sad mood caused a slight non-significant trend toward risk aversion. However, we find that participants with low levels of emotional contagion tend to make fewer risky choices after sad stimuli compared to neutral and joyful ones. A similar trend at the edge of significance was observed in participants with high levels of SS.

## Materials and Methods

### Participants

Eighty nine participants residing in Moscow were recruited to participate in the experiment. One participant was excluded from the analysis for not responding during the task. After removing this participant, the sample size was 88 (60 females) aged 16–45 years (mean 22.7 years, SD = 6.2). The local Ethics Committee of National Research University Higher School of Economics approved the study. All participants read and signed an informed consent prior to the experiment, and received a monetary reward for participation (see section “Procedure” for details).

Twenty additional participants (17 females) aged 18–35 years (mean 23.4 years, SD = 4.7) were recruited in order to validate the emotional effect of the stimuli. This pre-test was conducted through the online experiment platform, Pavlovia (Peirce et al., 2019). The pre-test participants received a flat fee of 200 RUB (≈$2.7, exchange rate $1 ≈ 75 RUB during the period of data collection). No participant took part in both portions of the experiment. All participants were recruited via social media platforms. Further details about the pre-test can be found in the Supplementary Table 1.

### Materials

#### Procedure

Each participant filled out an online demographic questionnaire before the experiment, stating their age, level of education, occupation, and history of any psychological or neurological diagnosis. In addition, they filled out two questionnaires. First, we applied the adventure seeking and experience seeking subscales of the SS scale (Zuckerman, 1979b; Egorova and Pyankova, 1992; for Russian adaptation, the internal consistency in the current study, α = 0.62). Second, we used the emotional contagion scale (Doherty, 1997) that we translated (the internal consistency in the current study, α = 0.75).

Upon arrival at the laboratory, the participants completed a practice session, containing 10 trials of the decision-making task. In the experimental design, at the beginning of each block, participants were asked to rate valence and arousal of their current mood on a 9-point Self-Assessment Manikins scale (Bradley and Lang, 1994). Afterward, participants watched four emotional video clips of the same condition (i.e., joyful, sad, or neutral). After each of the four clips, participants were asked to rate the valence and arousal of their current mood again. Once they watched the video clips, they performed 48 trials of the decision-making task for approximately 4 min. The participants were instructed to make each decision within 4 s, otherwise the decision was skipped and the experiment continued to the next trial. The described procedure was repeated three times, one for each emotional condition, the order of which was randomised. At the end of the experiment, three monetary outcomes – one from each block – were randomly chosen, summed, divided by the coefficient of 10 (unbeknownst to the participants) (Efimov and Ntoumanis, 2021), and paid to the participants on top of the flat fee of 500 RUB (≈$6.7) for participation.

#### Emotional Movie Clips

In the main experiment, the participants were presented with several pre-validated, emotional video clips which induced a joyful, sad, or neutral mood. FilmStim database (Schaefer et al., 2010) was used as a primary source of the emotional clips. Several clips with the highest scores for joy and sadness along with low scores for other emotions were chosen. The rest of the clips were chosen from popular films. Neutral clips included documentaries, urban and wildlife scenery, as well as a series of still pictures. During the validation process, participants were asked to watch each video clip and rate valence and arousal of their current mood and six basic emotions – sadness, joy, fear, anger, surprise, and disgust – on a scale from 1 to 9. The order of presentation of clips was randomised.

Ten joyful, 10 sad, and 12 neutral clips were pre-tested in total. Four clips with the highest scores for joy and sadness alongside low scores for other emotions were chosen for the two respective categories. Four clips with the lowest scores for joy and sadness were chosen for the neutral category (Supplementary Table 1).

We implemented four clips per category instead of one, in order to mitigate the potential caveat that something other than the hypothesised emotional effect (e.g., likeability of the character) would cause differences in findings given the naturalistic nature of the stimuli.

#### Decision-Making Task

**FIGURE 1:**
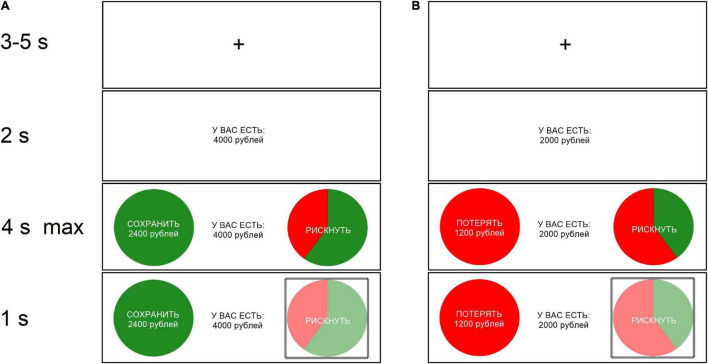
Sample trial framed as gain **(A)** and sample trial framed as loss **(B)**. Each trial started with the participants receiving an endowment (e.g., “You have 4000 rubles”). Then, two options were presented to the participant: a safe one (a full green or red circle) which offered them a proportion of the endowment for sure, and a risky one, which offered them to keep the full endowment with probability *p*. In the risky option, there was a 1-p probability of losing the whole endowment. The probability p was illustrated using a pie chart, in which the green-coloured slice indicated the probability to win and a red-coloured slice indicated the probability to lose. The gain and loss trials differed in the presentation of the safe option. In the gain frame, the safe option was formulated in terms of how much of the endowment was kept (e.g., “Keep 2400 rubles”), whereas in the loss frame, it was in terms of how much was lost (e.g., “Lose 1200 rubles”). After the participant made a decision, a square, semi-transparent box appeared around the selected option for 1 s before the next trial began.

The task was the same as in a previous study on mood induction (Stanton et al., 2014). At the beginning of each trial, participants were presented with a monetary endowment. Afterward, they had to choose between preserving a guaranteed proportion of the endowment versus gambling for the entire endowment while risking getting nothing, with various probabilities across trials. In half of the trials, the certain option was framed in terms of gains and in the other half of them it was framed in terms of losses (Figure 1). The endowment levels ranged from 2000 RUB (≈$27) to 8000 RUB (≈$107) with 2000 RUB increment. The probability of winning the gamble was 20, 40, 60, or 80% (see Supplementary Table 4 for the list of exact gambling options). Each possible combination of endowment and winning probability was presented once in each frame. The value of the certain option was matched with the expected value of the risky option in 32 of the 48 trials. In eight trials, the value of the certain option was higher, and in eight trials, it was lower than the expected value of the risky option. Following Stanton et al. (2014), no feedback about trial outcomes was given.

#### Data Analysis

In order to assess the emotional effects of the video stimuli, we calculated how the participants’ valence changed after each video condition. Specifically, the self-reported valence before each clip was subtracted from the self-reported valence after each clip and these differences were subsequently averaged within each condition. Therefore, the resulting values represent how much the videos of each condition changed the participants’ valence, on average, yielding the measure of mood induction. Due to the non-normal distribution of the self-reported valence (Shapiro–Wilk test, *p* < 0.05 for each domain), we compared the mood induction between conditions using a non-parametric Friedman test for dependent variables, followed up by pairwise Wilcoxon signed-rank tests.

To examine whether mood induction and framing affected risk taking, we conducted a two-way, repeated-measures analysis of variance taking Mood (three levels: sadness, neutral, joy) and Frame (two levels: gain, loss) as within-subjects factors (ANOVA, 3 moods × 2 frames). Risk taking (the dependent variable) in each session was estimated by calculating the proportion of times that individuals chose the risky option over the safe one. We also calculated the Pearson coefficient of correlation between the average change in self-reported valence and risk taking, separately for each mood condition. No significant correlation between the valence change and risk taking was found (Supplementary Figure 2). Using the absolute valence or arousal rating at the end of the clips instead of the average change in valence revealed no significant correlations.

To counter the possibility that the effect of mood and framing on risk taking is influenced by participants’ sensitivity to value information, we investigated the impact of biassed expected value on participants’ choices. To that end, we split all trials into three types depending on bias. Although, in most of the trials, the expected value of the safe option was equal to the expected value of the risky option (balanced trials), there were some trials where the expected value of the safe option was much greater than the expected value of the risky option (biassed trials in favour of the safe option) or vice versa (biassed trials in favour of the risky option). In order to assess the effect of mood and frame on risk taking, we additionally conducted two-way repeated measures ANOVA (3 moods × 2 frames), within each category of bias.

Furthermore, we investigated whether personality traits, such as SS and contagion, interact with the effect of mood induction and framing on risk taking. Participants were classified as “low” or “high” sensation seekers and as having “low” or “high” emotional contagion on the basis of whether they exceeded a median score on the relevant scales. We adopted this median split as it is common in experimental studies (e.g., Dar et al., 2019; Lin and Lee, 2021), yielding 43 participants as “low” and 45 “high” sensation seekers, and classifying 44 participants as having “low” emotional contagion and 44 as “high.” We performed a two-way, mixed ANOVA to evaluate the effect of Mood as a within-subjects factor (three levels: sadness, neutral, joy) and SS (two levels: high and low) as a between-subjects factor on risk-taking. The same was done for the emotional contagion trait. In both cases, there were no extreme outliers as assessed by the box plot method, and the data were normally distributed as assessed by the Shapiro–Wilk test (*p* > 0.05).

## Results

### Mood Induction

**FIGURE 2:**
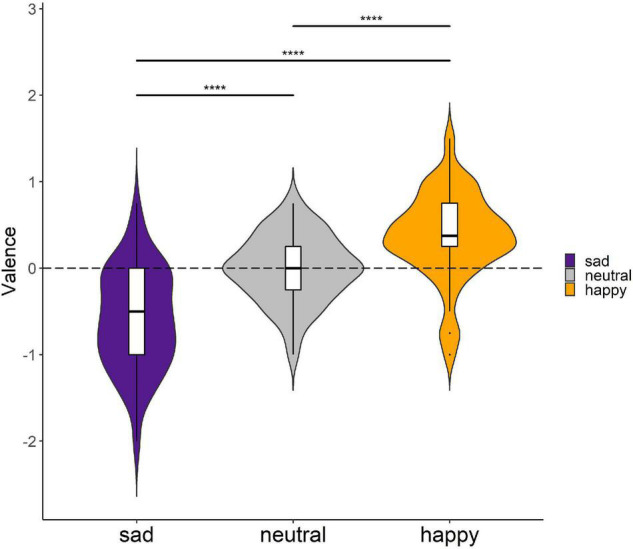
Changes in the participants’ valence after the joyful, sad, and neutral videos. A dashed horizontal line at 0 indicates no mood change. ****p < 0.0001.

A Friedman test revealed that there was a significant effect of video clip mood on participants’ changes in valence (*Q* = 87.6, *p* < 0.0001, effect size Kendall’s *W* = 0.498; Figure 2). Subsequent pairwise Wilcoxon signed-rank tests revealed statistically significant differences in valence change between joyful and sad [*W* = 67, Benjamini–Hochberg (BH) adjusted *p* < 0.0001; Benjamini and Hochberg, 1995], joyful and neutral (*W* = 550, BH adjusted *p* < 0.0001), and sad and neutral conditions (*W* = 299, BH adjusted *p* < 0.0001). Notably, the observed differences were all in the anticipated direction, i.e., sad videos reduced valence, joyful videos increased valence, and neutral videos had no effect.

### Effect of Mood Induction and Framing on Risk Taking

**FIGURE 3:**
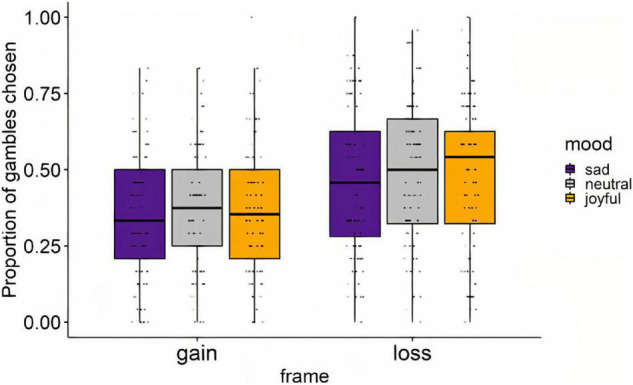
Risk taking (proportion of gambles chosen) by mood condition and frame. Dots represent individual subjects.

A two-way, repeated-measures ANOVA was then performed to evaluate the effect of framing and mood induction on the proportion of gambles chosen (Figure 3). The main effect of Frame was statistically significant [*F*(1,87) = 74.56, *p* < 0.0001, generalised eta-squared = 0.067], with participants opting for a risky option more in the loss frame than in the gain frame trials. However, the main effect of Mood was not significant [*F*(2,174) = 1.66, *p* = 0.193, generalised eta-squared = 0.002]. The interaction between Frame and Mood on risk taking was not significant [*F*(2,174) = 0.207, *p* = 0.813, generalised eta-squared < 0.001]. Planned pairwise comparisons were performed to assess the replicability of previous findings indicating differences in risk taking between the mood conditions (Stanton et al., 2014). Pairwise *t*-tests showed the trend that participants selected the risky option less frequently following sad versus neutral videos (*t* = −2.13, *p* = 0.034, BH adjusted *p* = 0.103), yet with the effect not reaching significance after *p*-value correction. No significant difference was found for the joyful versus neutral conditions (*t* = 0.513, *p* = 0.142, BH adjusted *p* = 0.213), nor between the joyful versus sad conditions (*t* = −1.47, *p* = 0.609, BH adjusted *p* = 0.609). Furthermore, no differences were found when the same pairwise comparisons were run within each frame category separately (Supplementary Table 2).

The “framing effect” has been defined in previous studies as the proportion of gambles chosen in the loss frame trials minus the proportion of gambles chosen in gain frame trials (Stanton et al., 2014). The framing effect did not differ significantly between different mood conditions, as assessed by a Friedman test (*Q* = 1.50, *p* = 0.473, Kendall’s *W* = 0.009).

Furthermore, the decision-making task contained some biassed trials, either in favour of the risky option or in favour of the safe option (see section “Decision-Making Task” and Supplementary Table 4). In the trials that were biassed in favour of the risky option, participants selected it 71.6% of the time, whereas in the trials that were biassed in favour of the safe option, participants selected the safe option with a frequency of 90.7%. In order to account for such floor and ceiling effects, we conducted a supplementary analysis by distinguishing these three categories of trials (Supplementary Figure 1).

The main effect of Frame was statistically significant in all three categories [unbiased trials: *F*(1,87) = 62.3, *p* < 0.0001; biassed trials in favour of the safe option: *F*(1,87) = 11.7, *p* = 0.0010; biassed trials in favour of the risky option: *F*(1,87) = 27.4, *p* < 0.0001]. However, the main effect of Mood was not significant for any of the three categories of bias [unbiased trials: *F*(2,174) = 2.86, *p* = 0.06; biassed trials in favour of the safe option: *F*(1.84,160) = 0.307, *p* = 0.717; biassed trials in favour of the risky option: *F*(1.82,158) = 2.09, *p* = 0.131]. Moreover, the interaction of Mood and Frame was non-significant for each bias type [unbiased trials: *F*(2,174) = 0.313, *p* = 0.732; biassed trials in favour of the safe option: *F*(1.61,140) = 0.522, *p* = 0.556; biassed trials in favour of the gamble option: *F*(2,174) = 1.49, *p* = 0.228].

The correlation between the two variables, self-reported valence and risk taking, failed to reach significance for all three conditions: joyful (Pearson’s correlation coefficient *r* = −0.09, *p* = 0.22), sad (*r* = −0.05, *p* = 0.51), and neutral (*r* = −0.08, *p* = 0.28, Supplementary Figure 2).

### The Effect of Mood Induction and Framing on Risk Taking in Relation to Personality Traits

**FIGURE 4:**
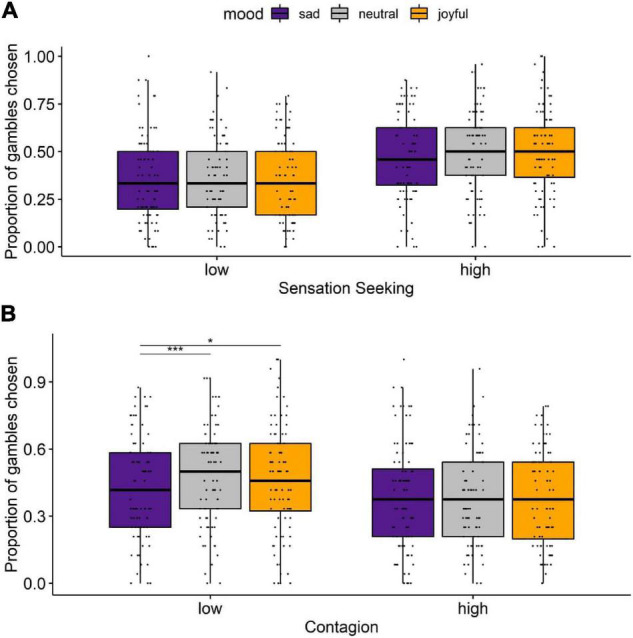
The effect of mood induction and framing on risk taking (proportion of gambles chosen), separately for low and high sensation seekers **(A)** and for individuals with low and high emotional contagion **(B)**. Dots represent individual subjects. Purple represents sad mood domain, grey represents neutral mood domain, and yellow represents joyful mood domain. *p < 0.05, ***p < 0.001.

We performed a two-way mixed ANOVA of risk taking with Mood as a within-subjects factor (sad, neutral, joyful) and SS as a between-subjects factor (high versus low, Figure 4A). Mood’s main effect was not significant [*F*(2,172) = 1.666, *p* = 0.192] while SS’s main effect was significant [*F*(1,86) = 12.234, *p* = 0.0007]. The two-way interaction between Mood and SS was not statistically significant [*F*(2,172) = 1.212, *p* = 0.300]. Further, we separately explored the effects of mood condition within low versus high sensation seekers. For high sensation seekers, the sad condition differed from both neutral (*t* = −2.14, *p* = 0.035, BH adjusted *p* = 0.064) and joyful (*t* = −2.05, *p* = 0.043, BH adjusted *p* = 0.064) conditions at the edge of significance (Figure 4A). No other pairwise comparison reached significance (Supplementary Table 5). We also compared framing effects by mood conditions separately for groups with low and high SS. The framing effect did not differ significantly between mood conditions in the group with low SS (*Q* = 1.05, *p* = 0.592, Kendall’s *W* = 0.014) and in the group with high SS (*Q* = 0.316, *p* = 0.316, Kendall’s *W* = 0.004).

We also performed a two-way mixed ANOVA to evaluate the effects of Mood as a within-subjects factor (sadness, neutral, joy) and Emotional Contagion as a between-subjects factor (high versus low) on risk taking (Figure 4B). The main effects of Mood and Emotional Contagion were not significant [Mood: *F*(2,172) = 1.709, *p* = 0.184; Emotional Contagion: *F*(1,86) = 3.304, *p* = 0.073]. The two-way interaction between Mood and Emotional Contagion was statistically significant [*F*(2,172) = 3.440, *p* = 0.034]. We also compared mood domains within groups with low and high emotional contagion scores. Specifically, in the low emotional contagion group, the sad condition significantly differed from both neutral (*t* = −4.20, *p* < 0.0001, BH adjusted *p* = 0.0002) and joyful (*t* = −2.47, *p* = 0.015, BH adjusted *p* = 0.023) conditions (Figure 4B). No other pairwise comparison reached significance (Supplementary Table 6). Moreover, we compared framing effects by mood conditions separately for groups with low and high emotional contagion. The framing effect did not differ significantly between different mood conditions in the group with low emotional contagion (*Q* = 0.05, *p* = 0.975, Kendall’s *W* < 0.001) and in the group with high emotional contagion (*Q* = 3.03, *p* = 0.220, Kendall’s *W* = 0.034).

For both personality traits (i.e., SS and emotional contagion), we repeated the above analysis, by also taking the framing into account, but no significant interaction was found between Frame, Mood, and the corresponding personality trait on risk taking (Supplementary Figure 3).

## Discussion

In the present study, using a within-subjects design, we modulated the participants’ emotional valence by presenting joyful, sad, and neutral naturalistic videos. Our findings demonstrate that joyful mood, induced by validated stimuli, did not affect economic decision making contrary to previous studies (Schulreich et al., 2014; Stanton et al., 2014; Pastwa and Imbir, 2019; Hareli et al., 2021), while induced sad mood resulted in a slight non-significant trend of risk aversion. Limiting the analysis to trials where the safe and the risky options have the same expected value did not reveal a significant main effect of mood on risk taking. However, according to our results, individuals scoring high on the SS scale and those scoring low on the emotional contagion scale showed risk-averse tendencies in the sad condition compared to neutral and joyful conditions.

Stanton et al. (2014) used a between-subjects design, leaving room for potential confounds such as differences across the groups (various personality traits might influence the data in such a design) and undermine generalisability of the findings. In order to replicate the between-subjects analysis of Stanton et al. (2014), we also extracted the data corresponding only to the first block for every participant and compared the valence changes, as well as the effect of induced moods on risk taking between participants. This revealed no significant effect of mood (Supplementary Figure 4).

On the other hand, framing influenced participants’ economic decision making. Our participants gambled more often in trials framed as losses compared to trials framed as gains (see Figure 1), regardless of their emotional state. This finding supports the framing effect idea as pioneered by Tversky and Kahneman (1981), according to which individuals tend to avoid risk in situations framed positively, but seek risks when a negative frame is presented. Our study thus joins a list of earlier studies that found empirical evidence for the framing effect (Stanton et al., 2014; Steiger and Kühberger, 2018). Moreover, our results support the findings of Stanton et al. (2014) that the framing effect is not affected by the induced emotional state.

Most studies investigating the effect of induced moods on risk taking have not included participants’ personality traits in their analysis. We consider this an important limitation since personality factors, such as SS and emotional contagion, can have an interaction with framing (Gabriel and Williamson, 2010) and mood (Kuang et al., 2019) in the context of risk taking. Such moderation effects of personality traits have been investigated in the past (see Kuvaas and Kaufmann, 2003; Garon and Moore, 2007; Sundqvist and Wennberg, 2014; Charpentier et al., 2016; Schulreich et al., 2016, 2020). In another instance, it was argued that negative affect and fun-seeking personality traits have independent effects on risky decision making (Suhr and Tsanadis, 2007).

The main effect of SS on risk taking proved to be statistically significant, despite its non-significant interaction with Mood and Frame. This suggests that sensation seekers take more economic risks than sensation-averse individuals, regardless of their emotional state and the way the decision is framed. This is in line with previous findings showing that individuals who score higher on SS take monetary risks (Kirkcaldy and Furnham, 1993) and gamble more (Kassinove, 1998).

In addition, our analysis revealed that induced sadness had a trend at the edge of significance toward lessening risk taking in people scoring high on the SS scale compared to joyful and neutral mood states in a manner that was not specific to particular framing. Although this result has to be considered with caution due to the insignificance and overall insignificant interaction of mood and SS, it suggests that risk taking is susceptible to alteration by sadness in a pronounced way in sensation seekers.

Unexpectedly, the effect of decreased risk taking in induced sad mood relative to the neutral or joyful mood was significant for the group with low emotional contagion. Although this finding has to be considered with caution, if this effect is proven significant in future studies, it may provide new insights about the influence of interaction of emotional contagion and sadness on risk taking.

To our knowledge, this study is the first to explore the interaction between the personality trait of emotional contagion and risk taking. Since the current design was founded on mood induction based on emotional naturalistic stimuli, we hypothesised that individuals’ emotional contagion will have an effect on how they perceive the stimuli and how they subsequently make economic decisions. Contrary to our hypothesis, the participants’ valence changes following watching the videos did not interact with their emotional contagion scores (Supplementary Figure 5). We conducted such an analysis regarding emotional contagion but not SS, since the former could, by definition, explain individuals’ reflexive production of the same emotions displayed in the stimuli.

Certain limitations of this study need to be taken into account. First, we induced positive, negative, and neutral moods in the same participants within a short experimental session of approximately 1 h. This might have caused some carry-over effects in the second and third blocks of the experiment. This limitation is unlikely to be critical, however, as the effect of each video domain on the proportion of gambles chosen was compared between situations where each possible carry-over effect was present versus absent, and it did not reach significance (Supplementary Figure 6). Second, the experimental task used in the present study represents the simplest decision-making task, i.e., individuals choose between two available options: a certain/safe and a risky one. Provided that many real-life decisions exist within a much richer context, the results need to be interpreted and generalised with caution.

In sum, our findings showed that sadness had a trend at the edge of significance (uncorrected *p* = 0.035, BH adjusted *p* = 0.064) toward lessening risk taking only in sensation-seekers and highly significantly reduced risk taking only in participants that were less susceptible to emotional contagion. These results suggest that processing sadness might significantly vary across the population and highlight the importance of including measures of personality traits in future studies of induced mood states effects on risk taking.

## Data Availability Statement

The original contributions presented in the study are publicly available. This data, as well as minimal code to reproduce the main findings, can be found here: https://osf.io/7nxce/ (Efimov and Ntoumanis, 2021).

## Ethics Statement

The studies involving human participants were reviewed and approved by the Ethics Committee of National Research University Higher School of Economics. Written informed consent to participate in this study was provided by the participants’ legal guardian/next of kin.

## Author Contributions

VKl, IJ, NK, AS, and KP contributed to the design of the study. OK and DK collected the data. KE, IN, and KP analysed the data. All authors contributed to the writing up of the manuscript and approved the final version of the manuscript.

## Conflict of Interest

The authors declare that the research was conducted in the absence of any commercial or financial relationships that could be construed as a potential conflict of interest.

## Publisher’s Note

All claims expressed in this article are solely those of the authors and do not necessarily represent those of their affiliated organizations, or those of the publisher, the editors and the reviewers. Any product that may be evaluated in this article, or claim that may be made by its manufacturer, is not guaranteed or endorsed by the publisher.
