# Supplementary material for: Impact of Induced Moods, Sensation Seeking, and Emotional Contagion on Economic Decisions Under Risk
Source: Front Psychol. 2022 Jan 5;12:796016. doi: 10.3389/fpsyg.2021.796016 (PMC8766662; doi:10.3389/fpsyg.2021.796016)
Supplement: Supplementary file 2 [file Data_Sheet_2.PDF]

Supplementary Figure 2

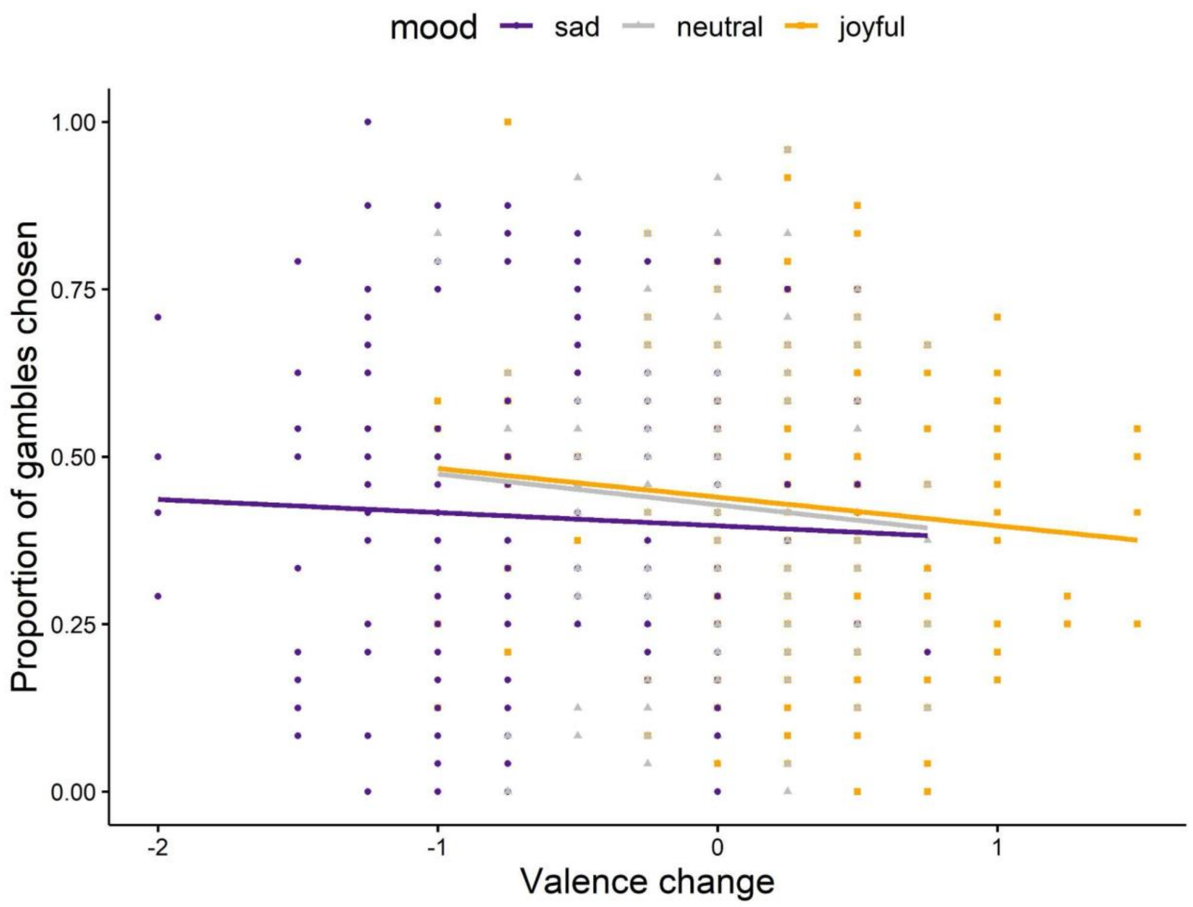

Supplementary Figure 2. Correlation between proportion of gambles chosen and changes in the self-reported valence after the videos of each mood domain. In none of the three domains, the correlation of the two variables of interest was found significant (sad: r = -.05, p = .51; neutral: r = -.081, p = .28; joyful: r = -.092, p = .22).
