# Supplementary material for: Impact of Induced Moods, Sensation Seeking, and Emotional Contagion on Economic Decisions Under Risk
Source: Front Psychol. 2022 Jan 5;12:796016. doi: 10.3389/fpsyg.2021.796016 (PMC8766662; doi:10.3389/fpsyg.2021.796016)
Supplement: Supplementary file 3 [file Data_Sheet_3.PDF]

Supplementary Figure 3

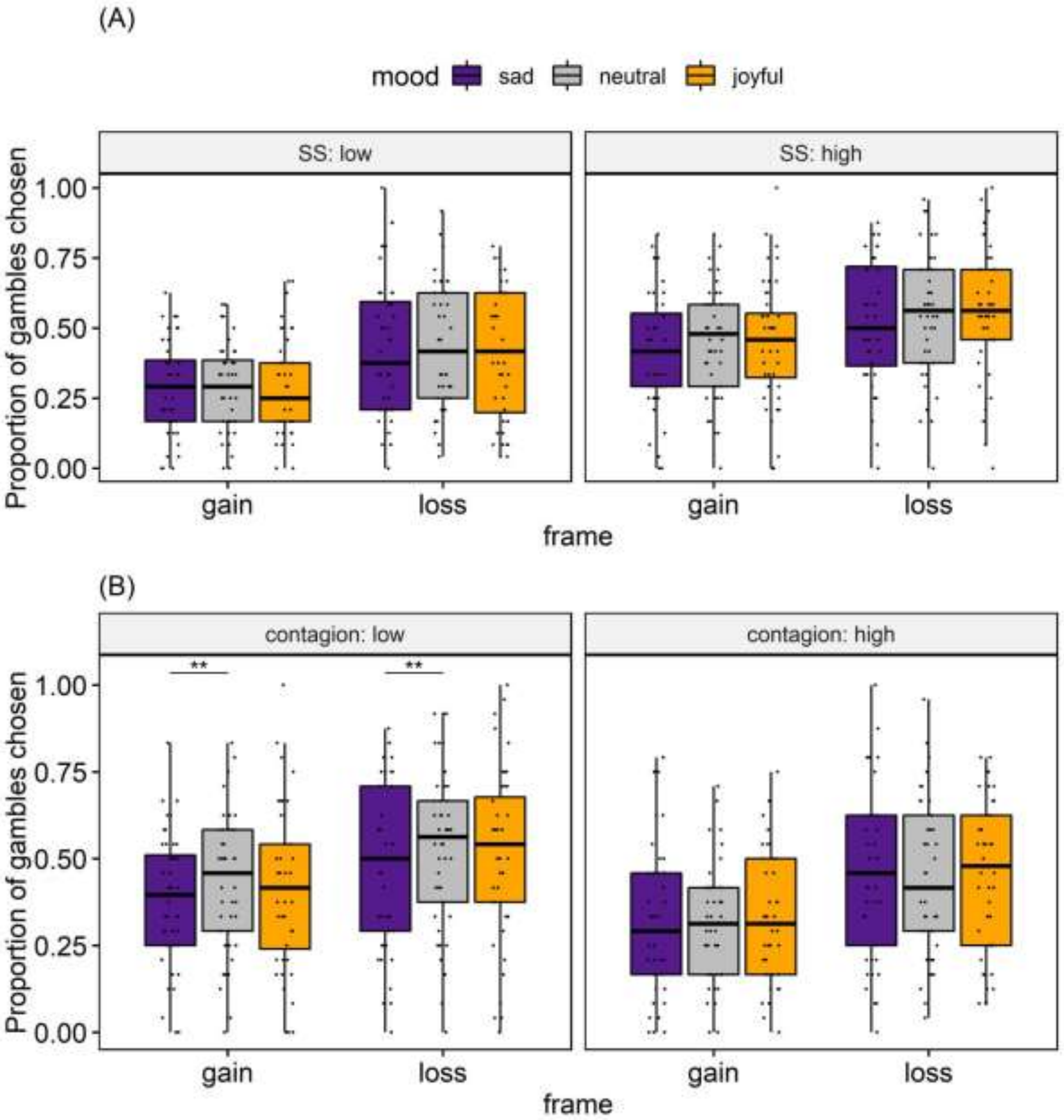

Supplementary Figure 3. The effect of mood induction and framing on risk-taking, separately for low and high sensation seekers (A) and for low and high contagious individuals (B). Dots represent individual subjects. Dots represent individual subjects. Purple represents sad mood domain, grey - neutral mood domain, yellow - joyful mood domain. For each personality trait (i.e. sensation seeking and contagion), we performed a three-way mixed ANOVA to evaluate the within-subjects effect of Mood and Frame and the between-subjects effect of the corresponding personality trait on risk taking. The three-way interaction failed to reach significance in both cases (Sensation Seeking: $F(2,172)=0.442$, $p=.644$; Contagion: $F(2,172)=0.344$, $p=.709$).
