# Supplementary material for: Impact of Induced Moods, Sensation Seeking, and Emotional Contagion on Economic Decisions Under Risk
Source: Front Psychol. 2022 Jan 5;12:796016. doi: 10.3389/fpsyg.2021.796016 (PMC8766662; doi:10.3389/fpsyg.2021.796016)
Supplement: Supplementary file 4 [file Data_Sheet_4.PDF]

Supplementary Figure 4

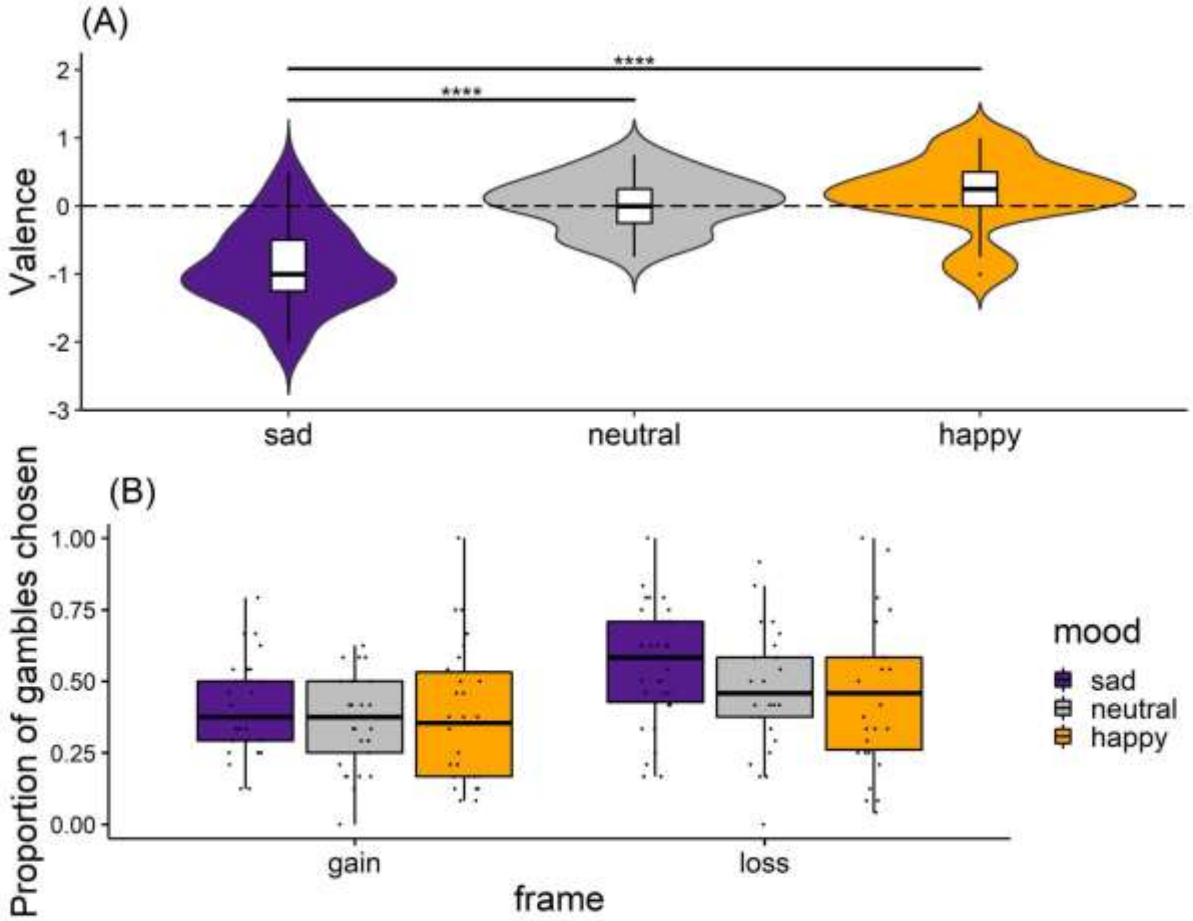

Supplementary Figure 4. (A) Replication of Figure 1, but including data only from the first block. A Kruskal-Wallis test showed that there is a significant effect of Mood on valence change (W=37.5, p<.0001, effect size = 0.413). This was followed by a post-hoc test, revealing a significant difference between sad and neutral (Dunn test statistic = 4.74, p<.0001), as well as between sad and joyful (Dunn test statistic = 5.72, p<.0001). The valence change after the joyful and neutral stimuli did not differ significantly (Dunn test statistic = 0.927, p=0.354). **(B)** Replication of Figure 2, but including data only from the first block. This is to illustrate the effect of induced moods on risk-taking in a between-subjects approach, as in Stanton et al., 2014. A mixed-ANOVA showed that the interaction between Mood and Frame was not significant (F(2,86)=1.886, p=0.158, ges=0.005). The main effect of Frame was statistically significant (F(1,86)=57.179, p<.0001, ges=0.074), so that the proportion of gambles chosen was higher in trials framed as loss. Despite the non-significant interaction, we performed post-hoc tests to replicate the results of Stanton et al., 2014, which nevertheless showed no significant pairwise differences between different moods, in none of the framing categories.
