# Supplementary material for: Impact of Induced Moods, Sensation Seeking, and Emotional Contagion on Economic Decisions Under Risk
Source: Front Psychol. 2022 Jan 5;12:796016. doi: 10.3389/fpsyg.2021.796016 (PMC8766662; doi:10.3389/fpsyg.2021.796016)
Supplement: Supplementary file 5 [file Data_Sheet_5.PDF]

Supplementary Figure 5

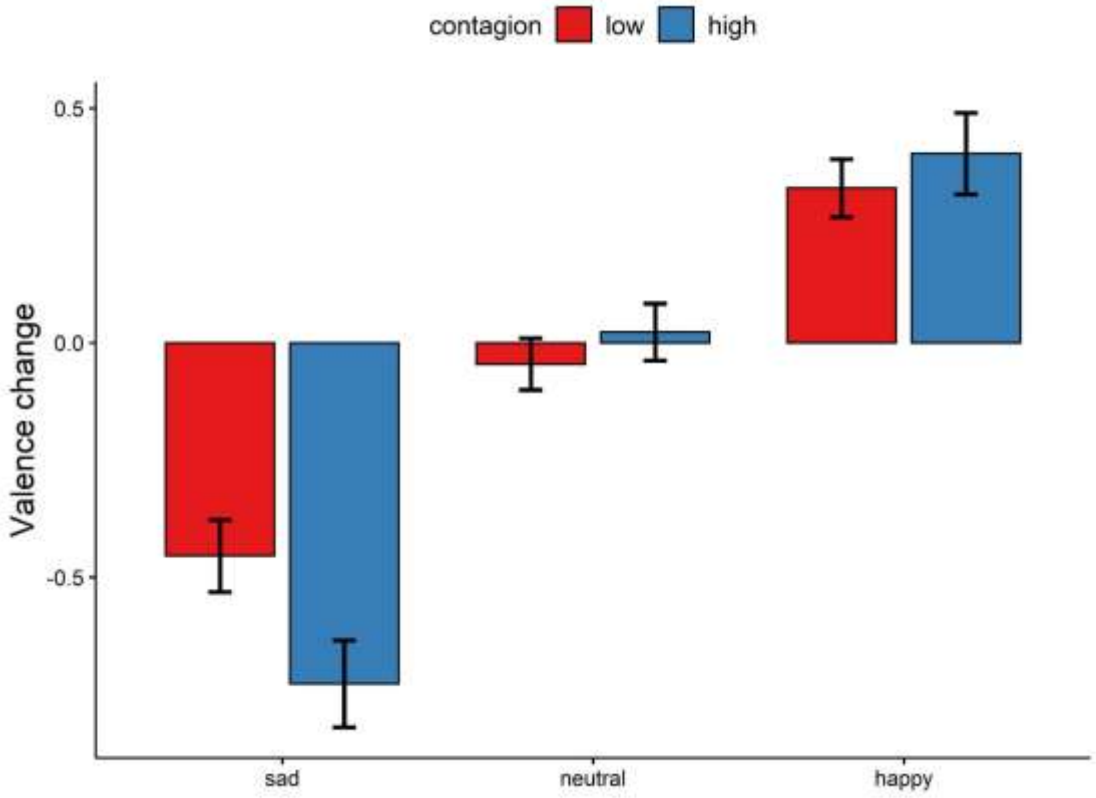

Supplementary Figure 5. The average valence changes after the joyful, sad, and neutral videos, for individuals scored “low” or “high” in the emotional contagion scale. As expected, highly contagious participants were more strongly affected by the emotional videos, yet with the interaction between Mood and Contagion on valence change not being significant (Wald-Type statistic=5.024, p=.081). This was assessed by a non-parametric mixed ANOVA, as implemented in the nparLD R package (Noguchi et al., 2012), since the data was not normally distributed (Shapiro-Wilk test, p<.05).
