# Supplementary material for: Impact of Induced Moods, Sensation Seeking, and Emotional Contagion on Economic Decisions Under Risk
Source: Front Psychol. 2022 Jan 5;12:796016. doi: 10.3389/fpsyg.2021.796016 (PMC8766662; doi:10.3389/fpsyg.2021.796016)
Supplement: Supplementary file 6 [file Data_Sheet_6.PDF]

Supplementary Figure 6

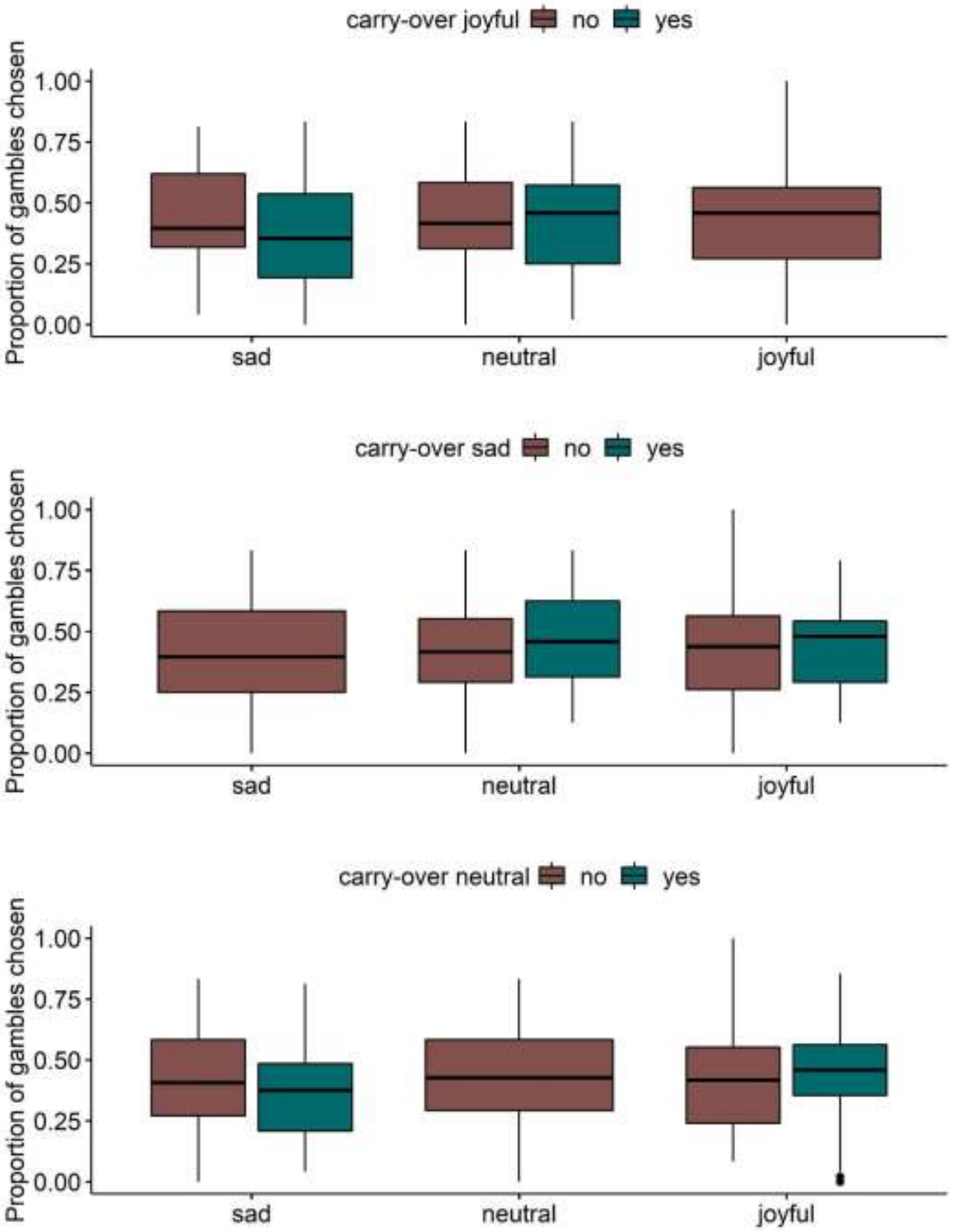

Supplementary Figure 6. Dummy variables were created denoting whether domain X preceded domain Y, for X,Y=”sad”,”neutral”,”joyful”. Then, the effect of each video domain on the proportion of gambles chosen was compared between situations where each possible carry-over effect was present vs. absent. In order to assess the statistical significance of the differences, we conducted pairwise t-tests. None of the three carry-over effects was significant at the level of α=0.05.
