# Supplementary material for: Impact of Induced Moods, Sensation Seeking, and Emotional Contagion on Economic Decisions Under Risk
Source: Front Psychol. 2022 Jan 5;12:796016. doi: 10.3389/fpsyg.2021.796016 (PMC8766662; doi:10.3389/fpsyg.2021.796016)
Supplement: Supplementary file 7 [file Data_Sheet_7.PDF]

Supplementary Table 1

Stimuli validation results. During the validation process participants were asked to watch 15 video clips, randomly selected from the list below, and rate their arousal on a scale from 1 to 9, as well as their six basic emotions on a scale from 1 to 5. The randomization was done so that each video was presented to 20 participants. The selection was done as follows: For the "joyful" condition we selected the videos with the highest rating in Joy, while the ratings in Sadness, Anxiety, Fear and Disgust were minimal. The selected joyful videos are indicated in the table with a golden background. For the "sad" condition we selected the videos with the highest rating in Sadness, while the ratings in Joy and Arousal were minimal. The selected sad videos are indicated in the table with a purple background. For the "neutral" condition we selected videos with minimal ratings in Joy and Sadness, while the rating in Interest was moderated. The selected neutral videos are indicated in the table with a gray background.

Clip	arousal	anger	anxi-ety	disgust	fear	inte-rest	joy	sadness	sur-prise
The visitors	4.84	1.71	1.25	1.21	1.6	5.2	3.35	1.36	4.3
Benny and Joon	5.17	1.39	1.84	1.53	1.41	5.54	5.16	1.5	4.52
Life is Beautiful	5.79	2.52	3.98	2.46	2.7	5.29	2.78	4.93	4.28
When Harry met Sally	5.35	2.19	2.22	2.06	1.78	4.92	4.5	1.62	4.46
Baraka	3.06	1.2	1.81	1.67	1.51	4.1	2.16	1.31	3.07
About endlessness	3.2	1.49	1.86	1.58	1.37	4	1.95	2.86	2.83
Used by others 1	2.61	1.73	1.62	1.49	1.31	2.79	1.89	1.36	2.44
Used by others 2	3.21	1.18	1.51	1.2	1.38	2.72	2.88	1.62	1.66
Walk	2.34	1.2	1.59	1.21	1.39	2.67	1.86	1.31	1.67
City of Angels	5.09	1.61	3.72	1.74	2.55	3.91	1.3	4.79	2.86
Dangerous minds	5.41	1.58	2.6	1.41	1.7	4.42	1.39	5.11	1.98

Dead man walking	6.12	3	4.99	4.57	3.87	4.87	1.26	4.52	3.43
Philadelphia	4.78	2.01	2.92	1.56	1.82	4.57	2.01	3.9	2.75
Three brothers	4.32	1.68	1.98	2.07	1.75	4.89	3.54	1.76	3.05
Baraka 2	2.58	1.26	2.05	1.25	1.65	3.46	2.61	1.45	1.75
How its made	4.39	1.26	1.29	1.13	1.25	5.66	3.1	1.15	3.62
Life is beautiful 2	6.02	3.13	4.95	2.29	3.54	5.28	2.11	5.71	2.63
What men talk about	3.83	1.42	1.39	1.7	1.13	4.75	4.29	1.24	3.5
Ivan Vasilievich	4.5	1.19	1.37	1.21	1.17	4.42	4.23	1.14	2.48
Den radio	4.03	2.25	2.16	2.83	1.73	4.29	3	1.44	3.03
Hotel Grand Budapest	3.81	1.4	2.03	1.48	1.31	4.44	3.09	1.56	2.89
Bruce	4.29	2.1	1.42	3.21	1.35	4.15	3.82	1.63	2.8
Manhattan	1.64	1.07	1.16	1.07	1.07	2.23	1.39	1.15	1.62
Wildlife	4.3	1.43	2.72	1.58	1.97	4.9	3.34	2.22	3.6
Boats	3.19	1.32	2.46	1.19	1.82	4.29	2.58	1.23	3.83
Gallery	1.97	1.63	1.44	1.34	1.05	2.14	1.39	1.25	1.47
Yamal	3.51	1.1	2.45	1.2	1.47	4.41	2.99	1.88	3.36
Leviafan	5.66	3.08	4.6	3.18	3.29	4.32	1.41	5.1	2.66
Aritmija	5	2.51	4.67	2.4	2.94	3.98	1.57	4.45	2.43
Boy without a leg	4.99	2.33	2.8	1.59	1.51	3.73	3.36	4.66	3.53
Mothers grave	5.22	1.56	2.38	2.03	2.15	3.7	1.47	5.27	2.47
Interstellar	5.12	1.54	3.34	1.41	2.21	5.24	1.44	5.04	3.03

Note: The number in the cell represents the average rating of the particular emotion, across 20 individuals.
