# Supplementary material for: Impact of Induced Moods, Sensation Seeking, and Emotional Contagion on Economic Decisions Under Risk
Source: Front Psychol. 2022 Jan 5;12:796016. doi: 10.3389/fpsyg.2021.796016 (PMC8766662; doi:10.3389/fpsyg.2021.796016)
Supplement: Supplementary file 8 [file Data_Sheet_8.PDF]

Supplementary Table 2

Post-hoc tests to assess the pairwise differences in risk-taking between mood domains, within each Frame category (gain, loss).

Frame	Mood 1	Mood 2	T-statistic	p-value	BH adjusted p-value
gain	sad	neutral	- 1.62	0.109	0.25
gain	sad	joyful	- 1.39	0.167	0.25
gain	neutral	joyful	0.07	0.944	0.944
loss	sad	neutral	- 1.40	0.166	0.498
loss	sad	joyful	- 0.71	0.479	0.507
loss	neutral	joyful	0.67	0.507	0.507

Note: ges stands for generalized eta squared.
