# Supplementary material for: Impact of Induced Moods, Sensation Seeking, and Emotional Contagion on Economic Decisions Under Risk
Source: Front Psychol. 2022 Jan 5;12:796016. doi: 10.3389/fpsyg.2021.796016 (PMC8766662; doi:10.3389/fpsyg.2021.796016)
Supplement: Supplementary file 9 [file Data_Sheet_9.PDF]

Supplementary Table 3

Number of participants per mood domain order.

Order	# Participants
joyful-sad-neutral	19
joyful-neutral-sad	11
sad-joyful-neutral	20
sad-neutral-joyful	10
neutral-joyful-sad	19
neutral-sad-joyful	9
