# Supplementary material for: Impact of Induced Moods, Sensation Seeking, and Emotional Contagion on Economic Decisions Under Risk
Source: Front Psychol. 2022 Jan 5;12:796016. doi: 10.3389/fpsyg.2021.796016 (PMC8766662; doi:10.3389/fpsyg.2021.796016)
Supplement: Supplementary file 10 [file Data_Sheet_10.PDF]

Supplementary Table 4

Exact gambles used in the decision-making task. The Safe option (last column) represents the number displayed in the safe option (either framed as gain or loss – first column). The third column represents the probability by which subjects could win the gamble and earn the whole endowment.

Exchange rate during the data collection period $1 ≈ 75 RUB

Framing	Endowment (rubles)	Probability to win the gamble	Safe option (rubles)
gain	2000	0.4	800
gain	2000	0.8	1600
gain	4000	0.2	800
gain	4000	0.4	1600
gain	6000	0.4	2400
gain	6000	0.8	4800
gain	8000	0.6	4800
gain	8000	0.8	6400
gain	4000	0.95	2000
gain	6000	0.95	3000
gain	2000	0.05	1000
gain	6000	0.05	3000
loss	2000	0.2	1600
loss	2000	0.4	1200
loss	4000	0.2	3200
loss	4000	0.6	1600
loss	4000	0.8	800
loss	6000	0.8	1200
loss	8000	0.2	6400
loss	8000	0.4	4800
loss	2000	0.95	1000
loss	6000	0.95	3000
loss	6000	0.05	3000
loss	8000	0.05	4000
gain	2000	0.2	400
gain	2000	0.6	1200
gain	4000	0.6	2400
gain	4000	0.8	3200
gain	6000	0.2	1200
gain	6000	0.6	3600
gain	8000	0.2	1600
gain	8000	0.4	3200
gain	2000	0.95	1000

gain	8000	0.95	4000
gain	4000	0.05	2000
gain	8000	0.05	4000
loss	2000	0.6	800
loss	2000	0.8	400
loss	4000	0.4	2400
loss	6000	0.2	4800
loss	6000	0.4	3600
loss	6000	0.6	2400
loss	8000	0.6	3200
loss	8000	0.8	1600
loss	4000	0.95	2000
loss	8000	0.95	4000
loss	2000	0.05	1000
loss	4000	0.05	2000
