# Supplementary material for: Impact of Induced Moods, Sensation Seeking, and Emotional Contagion on Economic Decisions Under Risk
Source: Front Psychol. 2022 Jan 5;12:796016. doi: 10.3389/fpsyg.2021.796016 (PMC8766662; doi:10.3389/fpsyg.2021.796016)
Supplement: Supplementary file 11 [file Data_Sheet_11.PDF]

Supplementary Table 5

Post-hoc tests to assess the pairwise differences in risk-taking between mood domains, within each Sensation seeking level (low, high).

**Sensation Seeking**	**Mood 1**	**Mood 2**	**T-statistic**	**p-value**	**BH adjusted p-value**
low	sad	neutral	-0.757	0.451	0.676
low	sad	joyful	0.181	0.857	0.857
low	neutral	joyful	0.925	0.357	0.676
high	sad	neutral	-2.14	0.035	0.064
high	sad	joyful	-2.05	0.043	0.064
high	neutral	joyful	-0.137	0.891	0.891

Note: ges stands for generalized eta squared.
