# Supplementary material for: Impact of Induced Moods, Sensation Seeking, and Emotional Contagion on Economic Decisions Under Risk
Source: Front Psychol. 2022 Jan 5;12:796016. doi: 10.3389/fpsyg.2021.796016 (PMC8766662; doi:10.3389/fpsyg.2021.796016)
Supplement: Supplementary file 12 [file Data_Sheet_12.PDF]

Supplementary Table 6

Post-hoc tests to assess the pairwise differences in risk-taking between mood domains, within each Emotional contagion level (low, high).

Emotional Contagion	Mood 1	Mood 2	T-statistic	p-value	BH adjusted p-value
low	sad	neutral	-4.20	<0.0001	0.0002
low	sad	joyful	-2.47	0.015	0.023
low	neutral	joyful	1.11	0.268	0.268
high	sad	neutral	0.674	0.502	0.847
high	sad	joyful	0.181	0.857	0.857
high	neutral	joyful	-0.578	0.565	0.847

Note: ges stands for generalized eta squared.
